# A novel method for non-invasively detecting the severity and location of aortic aneurysms

**DOI:** 10.1007/s10237-017-0884-8

**Published:** 2017-02-21

**Authors:** Igor Sazonov, Ashraf W. Khir, Wisam S. Hacham, Etienne Boileau, Jason M. Carson, Raoul van Loon, Colin Ferguson, Perumal Nithiarasu

**Affiliations:** 1 College of Engineering, Swansea University, Bay Campus, Fabian Way, Swansea, SA1 8EN UK; 2 Brunel University London, Uxbridge, London, B8 3PH UK; 3 Al-Khwarizmi College of Engineering, Baghdad University, Baghdad, Iraq; 4 Department of Vascular Surgery ABMUHB, Morriston Hospital, Swansea, SA6 6NL UK

**Keywords:** Aneurysm detection, Experimental models, Numerical models, One-dimensional modelling, Systemic circulation, Waveforms

## Abstract

The influence of an aortic aneurysm on blood flow waveforms is well established, but how to exploit this link for diagnostic purposes still remains challenging. This work uses a combination of experimental and computational modelling to study how aneurysms of various size affect the waveforms. Experimental studies are carried out on fusiform-type aneurysm models, and a comparison of results with those from a one-dimensional fluid–structure interaction model shows close agreement. Further mathematical analysis of these results allows the definition of several indicators that characterize the impact of an aneurysm on waveforms. These indicators are then further studied in a computational model of a systemic blood flow network. This demonstrates the methods’ ability to detect the location and severity of an aortic aneurysm through the analysis of flow waveforms in clinically accessible locations. Therefore, the proposed methodology shows a high potential for non-invasive aneurysm detectors/monitors.

## Introduction

Cardiovascular disease is responsible for the death of over eight million people worldwide every year. Among these deaths, aortic aneurysms alone are responsible for more than 100,000 deaths, with about 6000 occurring in England and Wales as a result of rupture. An aortic aneurysm is a dilation of the aorta, usually exceeding the normal diameter by more than 50%. The abdominal aortic aneurysm (AAA) is the most prevalent type of aortic aneurysm, and it is often asymptomatic. As it increases in size, the aneurysm is more likely to rupture and becomes a life-threatening condition. The symptoms are rarely noticed before rupture of the aneurysm, which prompted the healthcare systems to investigate various screening programs based on ultrasound and MRI. A Multicentre Aneurysm Screening Study (MASS) was one of the first large screening programmes for AAA in the UK. The results from the MASS programme showed that detection of AAA reduced risk of death in a 4-year period from 0.33 to 0.19% (Ashton et al. 2002).

Opportunistic detection of asymptomatic AAAs during clinical examination is the most common way of diagnosis. An abdominal palpation has only a moderate overall sensitivity for detecting AAAs (unless they are large enough to warrant elective intervention), especially in overweight people (see for instance the work by Fink et al. (2000)). Symptoms usually only occur near to or at the point of rupture. Although scanning the elderly population for aneurysms provides an excellent opportunity to reduce potential mortalities, developing cheaper and faster methods is an important challenge for medical and biomedical engineering.

In a clinical setting, ultrasound (US) is currently the most practical, non-invasive and inexpensive modality in screening for and surveillance of AAA, with a sensitivity and specificity of more than 98% (see among others Ashton et al. (2002); Wilmink et al. (2002); Sprouse et al. (2004); Barkin and Rosen (2004); Walker et al. (2004); Fleming et al. (2005); Catalano and Siani (2005); Brekken et al. (2011)). Some common limitations of US diagnosis include: suboptimal imaging, attenuation and inaccurate measurements, often due to bowel gas, obesity, artery tortuosity and/or calcification. Inter-observer variability can also be a problem. The shape and size of the aneurysm can be determined most accurately by means of 3D CT or MRI (Sparks et al. 2002; Lee et al. 1984; Litmanovich et al. 2009; McBride et al. 2015) methods.

The goal of the paper is to develop cost-effective AAA detection methods based on accessible measurement and analysis of human pressure and/or velocity waveforms. These waveforms can be easily computed using a framework of 1D systemic circulation models.

One-dimensional systemic circulation models have been the topic of intense research over the last 10 years (Barnard et al. 1966; Hughes and Lubliner 1973; Avolio 1980; Formaggia et al. 1999; Franke et al. 2002; Urquiza et al. 2006; Alastruey et al. 2007; Steele et al. 2007; Mynard and Nithiarasu 2008; Blanco et al. 2012; Chen et al. 2013; Watanabe et al. 2013; Müller and Toro 2013, 2014; Blanco et al. 2015; Boileau et al. 2015; Huang and Muller 2015). Recent explosion of more robust and benchmarked models has demonstrated the accuracy and usefulness of these models in predicting various blood flow quantities. Therefore, it makes sense to investigate the applicability of such systemic circulation models for the non-invasive detection of aneurysms. Preliminary attempts as proposed in (Low et al. 2012) show very encouraging results and illustrate a clear link between aneurysm shape and changes in the flow waveform as it appears that the pulse reflections are pronounced in the presence of aortic aneurysms. A general analysis of reflections as a result of disturbances has been analysed and discussed in several publications (Khir et al. 2001; Hughes and Parker 2009). In the work by Swillens et al. (2008), an aneurysm detection method is proposed based on a reflection coefficient by measuring pressure and flow rate waveforms. The pressure waveforms may be measured via arterial tonometry or cuff pressure measurements obtained from the wrist or carotid. The arterial velocity may be measured with ultrasonic echography.

**Fig. 1:**
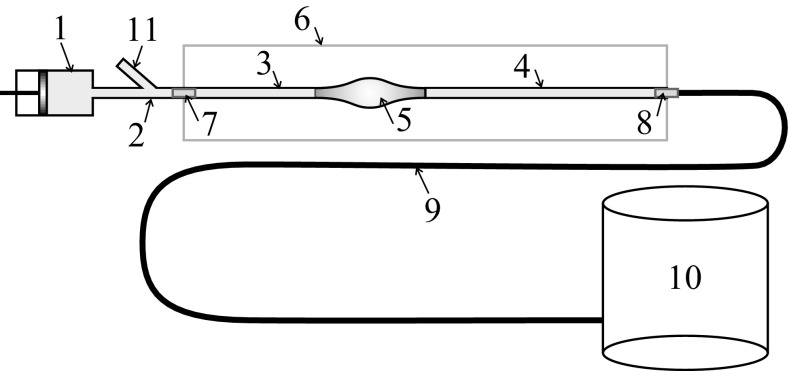
Laboratory set-up scheme: *1* pump, *2* inlet rigid tube, *3*, *4* main tube, *5* “aneurysm”, *6* transparent box, *7*, *8* fitting, *9* long tube, *10* reservoir, *11* branch for the pressure catheter

The modelling, analysis and measurements carried out in the past (Swillens et al. 2008; Low et al. 2012) clearly indicate that an accurate analysis and decomposition of measured pressure waveforms may provide an indication of existence of an aortic aneurysm. Thus, in the present work we determine and analyse the pressure waveform changes caused by reflection from an aneurysm. We study dependence of the reflection coefficient on aneurysm size and also compare the results with the pressure waveform in a healthy vessel. We also propose a method of determination of parameters indicating existence of an aneurysm depending on its location and its rate of change in cross-sectional area. To experimentally model a blood vessel with and without aneurysm, we have developed an experimental set-up, representing a part of the cardiovascular system. The arrangements and the parameters of this set-up are also used in the numerical simulations. After a thorough analysis of the experimental and numerical results, the indicative parameters of an aneurysm, identified from the simplified experimental and numerical model, are put into practice in a full systemic circulation model.

The paper is organized into the following sections. In Sect. 2, we describe the experimental set-up, method of measuring the pressure and velocity waveforms and the experimental results. In Sect. 3 the governing equations are presented and the numerical scheme is briefly described as well as the definition of the boundary conditions. The numerical results are compared with the experimental data in order to validate the numerical model. The wave analysis is discussed in Sect. 4. Here, the waveform is decomposed into forward and backward waves, and the formulation of the aneurysm indicators is presented. The method developed is then applied to the experimental and numerical data described in Sects. 2 and 3, respectively, and then, in Sect. 5, to a human arterial model network. Section 6 draws some conclusions and discusses the challenges and unresolved problems. Some auxiliary material is presented in Appendix including details of the numerical scheme (“Appendix 1”) and the waveform generated by the pump (“Appendix 2”).

## Experimental set-up and measurements

The experimental set-up includes a pump representing the heart and system of tubes characterizing the blood vessels. One of those tubes has a bulge to represent an aneurysm as shown in Fig. 1. Water is used as a working liquid. The pump, 1, generates pulses propagating through the system of tubes. It is directly connected to a rigid tube, 2, which through a rigid fitting, 7, is connected to the main tube, 3–4, located in a transparent box, 6. The tube segment with the artificial aneurysm, 5, is cut into the main tube. The outlet of the main tube through the fitting, 8, located in the wall of the box is connected to a 6.5-m-length pipe, 9, and its outlet is connected to a collecting reservoir, 10. This allows us to minimize the wave reflection. The rigid inlet tube has a branch, 11, through which the pressure measuring catheter is inserted.

**Fig. 2:**
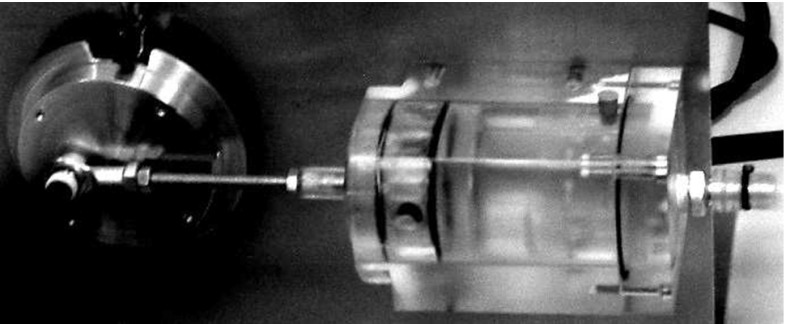
Pulse-generating pump

The main tube has an internal diameter of 17 mm, a wall thickness of 2 mm and a full length of 2 m. The measured Young modulus of the wall material is 2.8 MPa. The measured pulse wave propagation speed in the tube is 21 m/s. Its proximal to the aneurysm part is of 50 cm length. Pump 1 represents an engine rotating a wheel connected through the crank to the piston in a cylinder (see Fig. 2). The cylinder diameter is 5  cm. The piston stroke equals the double distance from the wheel centre to the joint with the crank (5  cm in length), which results in a stroke volume of approximately 100 $\hbox {cm}^3$. In the absence of the load from the piston and liquid, the pump and the wheel would rotate uniformly and move from one dead point to another, producing a half-sine pressure pulse. It should be noted that the load causes a non-uniform rotation of the wheel, which will affect the generated waveform. Such waveforms are analysed in Sect. 2. The pump is operated for only one cycle for every measurement in order to avoid reflections from the reservoir.

**Fig. 3:**
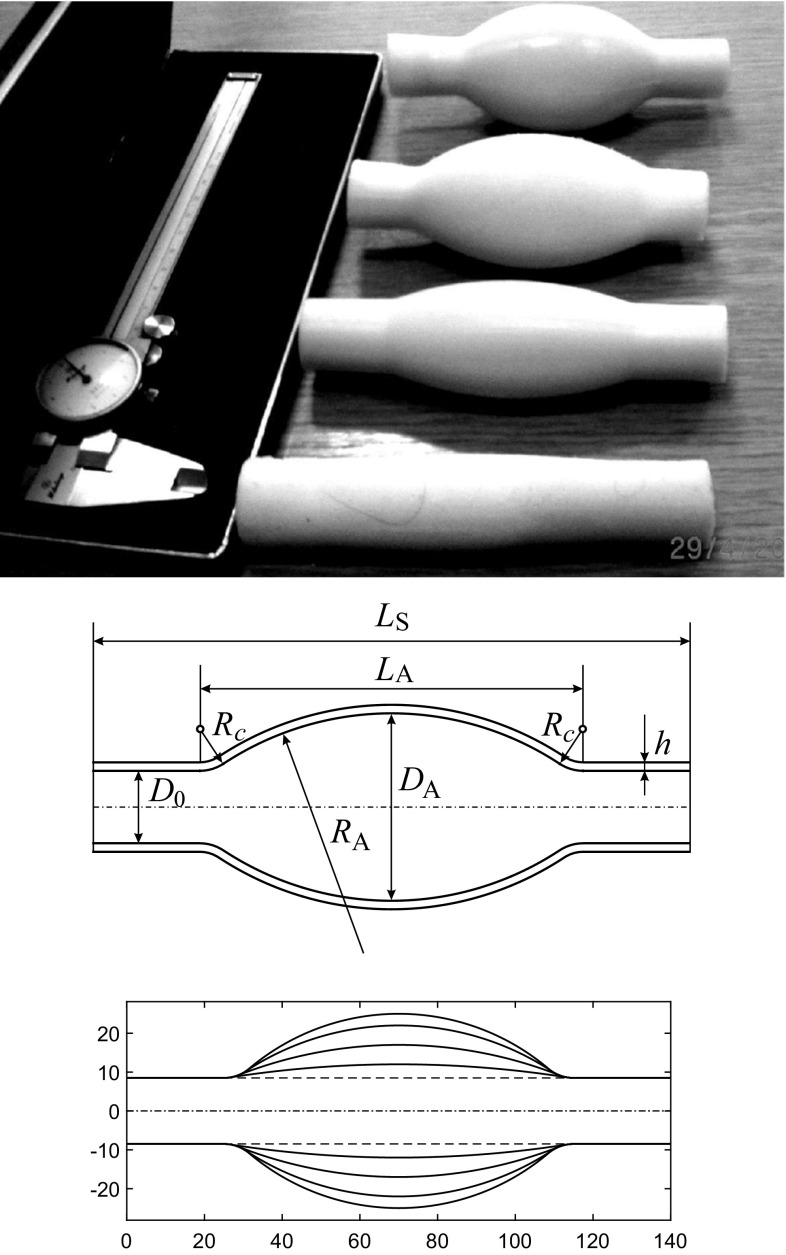
*Top* four tube segments with various aneurysm sizes. *Middle* geometric definition of the artificial aneurysms. *Bottom* Shapes of all the aneurysms considered. The dimensions are given in mm

Four different samples of artificial aneurysms of different sizes have been prepared, see Fig. 3, with maximal internal diameters of 24, 34, 44 and 50 mm, respectively.

**Fig. 4:**
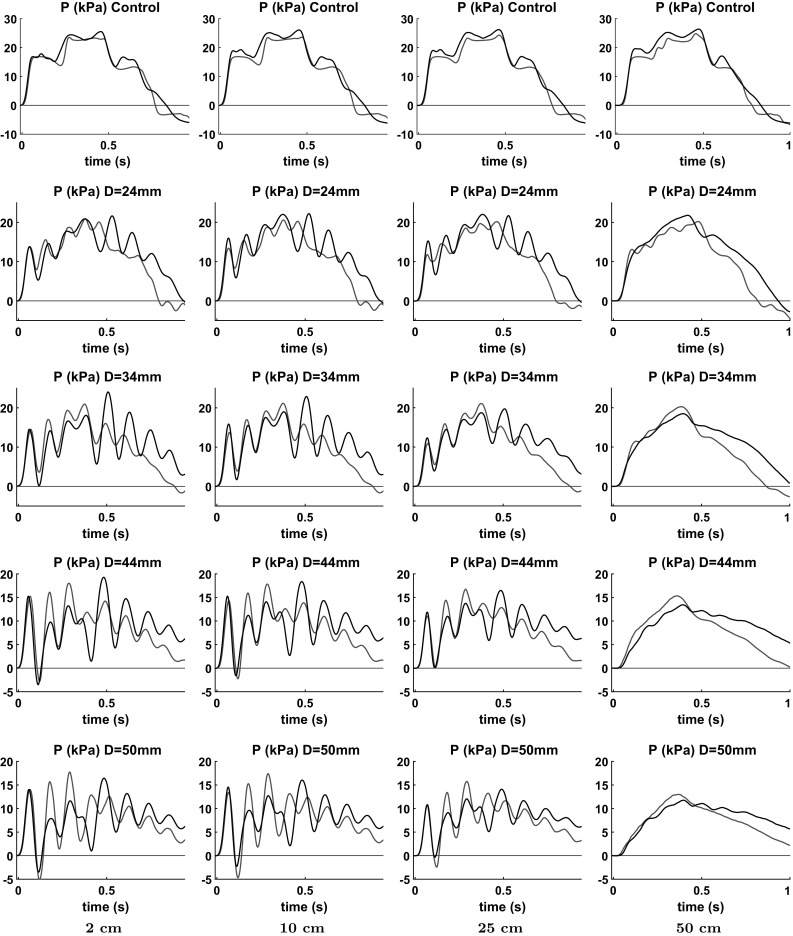
Comparison of experimental (*black*) and numerical (*red*) pressure waveforms. The aneurysm diameter is indicated in every plot. The site is indicated below every column of plots

The aneurysms are axially symmetric with the generatrix having the shape of a circular arc of radius $R_A$ smoothly conjugated to the constant area parts of the tube. Conjugating radii are $R_s = 10$ mm. The length of the aneurysm is $L_A = 9$ cm. It is located in the middle of the tube segment having a length $L_S = 14$ cm. Wall thickness, *h*, is kept uniform for all parts of the segment and $h=2$ mm. The elastic properties of the aneurysm tube segment are measured to be close to those of the main tube. Besides the aneurysm, similar experiments and measurements have been conducted on a control setting: a uniform main pipeline. The fitting is an approximately rigid tube with an internal diameter of 12 mm.

Pressure was measured using a 6 F pressure transducer-tipped catheter (Gaeltec, Scotland, UK), which was inserted into the tube through a Y-junction at the inlet of the tube or outlet of the tube. The flow rate was measured using ultrasound flow probes (Transonic System Inc, Ithaca, NY, USA). All measurements of the pressure and flow rate are taken in tube segment 3 shown in Fig. 1 in four sites: at a distance 2, 10, 25 and 50 cm from the inlet of the main tube.

Repeated measurements for every aneurysm in every site are taken from 8 to 15 for every combination of aneurysm size/site, resulting in more than 200 measurements overall. The basic sampling frequency is 500  Hz, but 1 kHz is also used in some measurements to ensure that pressure waveforms are properly captured. First, the measured waveforms are smoothed by convolution with a Gaussian function at a width of two time steps. As the recording is not synchronized with the pump, it is necessary to align all waveforms to start the main pulse at the same instant. Then the measured waveforms are averaged in the sample rate 500 Hz in order to decrease the influence of noise further.

The averaged measured pressure waveforms are presented in Fig. 4 indicated by black curves for all 20 cases: five aneurysm sizes (including the control tube) times four sites. We can observe strong oscillations at sites downstream from the inlet in the set-up with aneurysms. The further from the aneurysm the stronger the oscillations are. For those tests without an aneurysm (the first row of plots), we observe much smaller oscillations of similar amplitude in all the sites. Also we see that the greater aneurysm size the lower is the maximal pulse amplitude. The next stage of experimental data processing is described in Sect. 4.2.

## Numerical simulation

### Governing equations

A one-dimensional approximation is employed for the numerical simulation. This approximation is mainly based on Stergiopulos’s model (Stergiopulos et al. 1992) with some modifications adopted from Wang and Parker’s model (Wang and Parker 2004) to avoid large reduction in arterial material properties. In this approximation, the governing continuity and momentum equations can be written in the following form (Formaggia et al. 2001; Sherwin et al. 2003b; Mynard and Nithiarasu 2008)

1 $$\begin{aligned}&A_t+(Au)_x =0 \end{aligned}$$

2 $$\begin{aligned}&u_t+uu_x+\frac{1}{\rho } p_x-\frac{1}{\rho }\tau _x =0 \end{aligned}$$

where subscripts *t* and *x* denote the partial derivatives with respect to time *t* and coordinate along the pipe *x*, respectively; *A* is the cross-sectional area of the lumen; $u, \rho and p$ are, respectively, the average velocity, density and pressure of the liquid, whilst $\tau$ is the wall shear stress. Assuming cylindrical vessels and a Poiseuille flow we find:

3 $$\begin{aligned} \tau _x=-\frac{8\pi \mu u}{A} \end{aligned}$$

where $\mu$ is the dynamic viscosity of the liquid. Equations (1), (2) and (25) are supplemented with the commonly used pressure–area relation (Franke et al. 2002; Sherwin et al. 2003b; Mynard and Nithiarasu 2008)

4 $$\begin{aligned} p=p_{{\mathrm {ext}}}+\beta \left( \sqrt{A}-\sqrt{A_{0}}\right) \end{aligned}$$

where

5 $$\begin{aligned} \beta \equiv \left. \frac{d p}{d \sqrt{A}}\right| _{A=A_0} = \frac{\sqrt{\pi }hE^{\prime }}{A_{0}},\qquad E^{\prime }=\frac{E}{ 1-\sigma ^{2}}. \end{aligned}$$

Here *E* and $\sigma$ are the Young modulus and Poisson ratio for the wall material, respectively; $A_{0}$ is the initial tube inner area; $E'$ is an analogue of the Young modulus for plates and shells. Now Eqs. (1)–(4) form a closed system.

### Characteristic variables

Equations (1)–(25) can be rewritten in the standard form Low et al. (2012) as

6 $$\begin{aligned} \mathbf {U}_t+\mathbf {F}_x =\mathbf {S} \end{aligned}$$

where the variables term, $\mathbf {U}$, the flux term, $\mathbf {F}$, and the source term, $\mathbf {S}$, are

7 $$\begin{aligned} \mathbf {U}= \begin{bmatrix} A \\ u \end{bmatrix} ,\quad \mathbf {F}= \begin{bmatrix} uA \\ \displaystyle \frac{u^{2}}{2}+\frac{p}{\rho } \end{bmatrix} ,\quad \mathbf {S}= \begin{bmatrix} 0 \\ \displaystyle -\frac{8\pi \mu }{\rho }\frac{u}{A} \end{bmatrix} . \end{aligned}$$

The characteristic speed of the system of variables of the equations can be given as

$$\begin{aligned} \mathbf {A}= \begin{bmatrix} \lambda _{f}&0 \\ 0&\lambda _{b} \end{bmatrix} = \begin{bmatrix} u+c&0 \\ 0&u-c \end{bmatrix},\quad c^{2}=\frac{A}{\rho }\frac{\partial p}{\partial A}=\frac{ \beta }{2\rho }\sqrt{A} \end{aligned}$$

where *c* is the pulse wave speed in the tube. Under physiological flow conditions, it is known that the pulse wave speed is higher than the fluid velocity *u* (Formaggia et al. 2002, 2003; Sherwin et al. 2003a, c). Therefore, the system is strictly hyperbolic and subcritical with $\lambda _{f}>0$ and $\lambda _{b}<0$. The characteristic variables of the system are well known to have the form

8 $$\begin{aligned} w_{f}=u + 4c, \qquad w_{b}=u - 4c \end{aligned}$$

satisfying the first-order advection equations

9 $$\begin{aligned} (w_{f})_t + \lambda _{f}(w_{f})_x = 0,\qquad (w_{b})_t + \lambda _{b}(w_{b})_x = 0. \end{aligned}$$

Thus, characteristic $w_f$ describes a forward propagating wave (away from the heart) at a speed of $u+c$, whereas $w_f$ corresponds to a backward propagating wave (towards the heart) at a speed $u-c$ which can appear after reflection due to impedance changes in the cardiovascular system at bifurcations, aneurysms, and so on. The waves are nonlinear as *u* and *c* are amplitude dependent. If the characteristics are calculated, the physical variables can be easily restored via:

10 $$\begin{aligned} u = \frac{w_f + w_b}{2}, \quad p=p_{{\mathrm {ext}}}-\beta \sqrt{A_{0}}+ \frac{\rho }{8}\bigg (\frac{w_f - w_b}{2}\bigg )^2. \end{aligned}$$

With the characteristic variables, $w_f$ and $w_b$, appropriate boundary conditions may now be imposed. Moreover, because the two characteristic speeds of the system have opposite signs (Formaggia et al. 2002; Sherwin et al. 2003a; Formaggia et al. 2003), a single boundary condition needs to be specified each at the inlet and exit of a segment.

Variables $w_f$ and $w_b$ are actively employed in the numerical scheme in order to impose the boundary conditions. Equation (9) may be integrated to produce necessary forward and backward moving waves as

$$\begin{aligned} w_f^{n+1}(x^{\mathrm{in}})&= \bar{w}_f^{n+1}(x^{\mathrm{in}}) \\ w_b^{n+1}(x^{\mathrm{in}})&= w_f^{n+1}(x^{\mathrm{in}} - \lambda _b^n\Delta t). \end{aligned}$$

where superscript *n* denotes the *n*th time step for any variable and $\bar{w}_f$ is the characteristic variable computed from prescribed pressure or velocity.

### Boundary conditions

At the connections between tubes the continuity of the flow rate $Q = Au$ and the total pressure $p + \frac{1}{2} \rho u^2$ are imposed (Mynard and Nithiarasu 2008). The boundary condition at the outlet (reservoir) is not essential as we are dealing with a single pulse at a time period (less than 1 s) shorter than the time required for a wave to propagate from the pump to the reservoir and back (about 1 s). Nevertheless, we modelled the reservoir as a wide tube with a non-reflecting outlet boundary condition.

Most challenging is to determine the inlet conditions, i.e. the waveform produced by the pump. The wave generated by the pump can partly reflect from the aneurysm, producing a backward propagating wave, which may be reflected by the piston to form a forward propagating wave. The wave is generated only whilst the piston is in motion. Its waveform shape is determined completely by the piston velocity variation. The piston motion is subjected to load generated by the fluid resistance and motion. This load exists even in the absence of reflection (such case is considered in Appendix 2), but in the presence of the aneurysm, the reflected wave imparts an additional load on the piston motion. Thus, the generated waveform heavily depends on the aneurysm size. Note that when the piston is at rest, the backward velocity wave reflected from the aneurysm and reflected from the piston/rigid pipe should cancel each other at the point of contact. This not true, however, for the pressure. Thus, measuring the velocity waveform near the piston gives the actual piston motion. As the pipes connecting the pump and the main tube are rather rigid, the velocity close to the inlet of the main tube has almost the same velocity wave shape as that of the one generated by the piston but scaled by the piston/pipe area ratio.

**Fig. 5:**
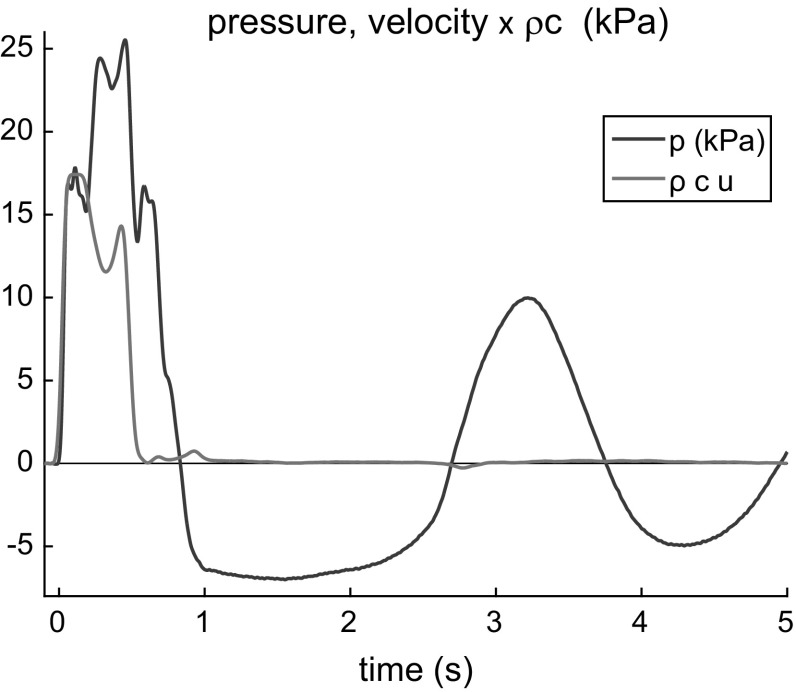
Pressure (*red*) and velocity (multiplied by $\rho c_0$) (*green*) waveforms at a distance of 2 cm from the inlet of the control tube

**Fig. 6:**
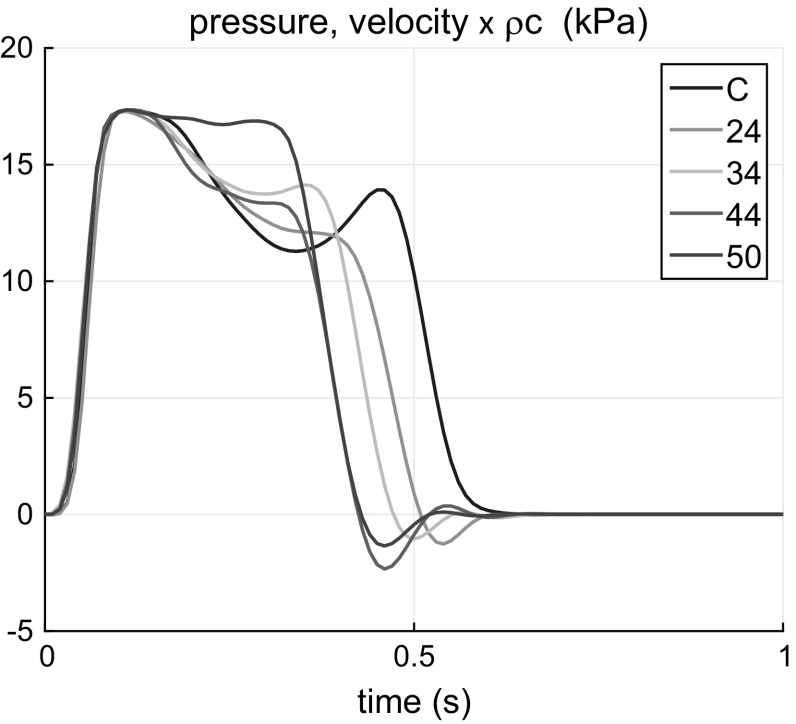
Initial waveforms for all four aneurysm sizes (indicated in the legend in mm) and the control tube (indicated by *letter C*)

One can see in Fig. 5 (green curve) that at 2 cm from the inlet, the velocity almost vanishes after $t>0.6$, i.e. when the piston stops its motion. At the same time, the pressure tends to have a low-frequency harmonic oscillation. Its period is about 2 s that is four times that of the wave propagation time from the piston to the reservoir.

Therefore, the velocity waveform measured at the interval $[0,0.6~\mathrm{s}]$ can be taken as the actual load adjusted waveform. As this wave is forward propagating one, the generated pressure waveform can be calculated by multiplying the velocity by $\rho c_0$ in the linear approximation. This pressure waveform is set as the inlet boundary condition in the numerical model. The somewhat uncommon two-humped shape is explained in “Appendix 1”. The load depends on the aneurysm size; therefore, for every aneurysm, the initial waveform is calculated independently. All the initial waveforms extracted from the experimental data and utilized in the numerical modelling are shown in Fig. 6.

### Numerical results

The results of the numerical simulations are shown in Fig. 4 by red curves. One can see that despite some discrepancies, the waveforms generally coincide. The frequency and the phase of oscillation are in better agreement than the amplitude. The fact is that the frequencies are determined by the time of wave propagation from the pump to a reflecting object (aneurysm, second fitting) and back. The amplitudes of the oscillations depend mainly on the reflection coefficients which are more sensitive to the set-up parameters, and it is not easy to measure some of them accurately. The discrepancies can also be attributed to the lack of the 1D model’s ability to accurately describe the process in the pump, losses in the tube, parameters of the fittings, transverse motion of the tube and other phenomena.

## Wave separation and reflection coefficient

### Transposition into forward and backward waves

There are two approaches on the separation of the waveform into forward and backward travelling waves. The first approach is based on the fact that nonlinear and viscous effects are relatively small. The second is based on the water-hammer equation and is valid for finite amplitude waves (wave intensity analysis) (Parker and Jones 1990; Khir et al. 2001; Swillens et al. 2008; Hughes and Parker 2009). We actively use the first approach by assuming that nonlinear and viscous effects are weak and can cause a noticeable change in the wave amplitude and shape only after propagation over a sufficiently large distance, i.e. beyond the domain of interest.

Now we set forth the basic statements of the linearized approach. Consider a tube of a constant cross section and with the constant wall thickness and stiffness. Also consider the case when the time averaged velocity is small compared to a typical velocity during pulse propagation. Finally let $p_{{\mathrm {ext}}}=\beta \sqrt{A_0}$, then $p=\beta \sqrt{A}$. Following the standard perturbation technique, we represent the area as $A = A_0 + \Delta A$ with $\Delta A\ll A_0$ and substitute them into (1), (25) and (4). Keeping the first-order terms after some manipulations we obtain:

11 $$\begin{aligned}&p_{t}+\rho c_{0}^{2}u_{x}=0 \end{aligned}$$

12 $$\begin{aligned}&u_{t}+\frac{1}{\rho }p_{x} = 0 \end{aligned}$$

where $c_0$ is the unperturbed wave speed given by

13 $$\begin{aligned} c_{0}^{2}=\frac{\beta \sqrt{A_{0}}}{2\rho }=\frac{hE^{\prime }}{2\rho a_0}. \end{aligned}$$

Here $$a_0$$ is a radius of the tube assuming that the tube is circular, $$A_0=\pi a_0^{2}$$ and $$E'=E/(1{-}\sigma ^2)$$. Eliminating $$u_t$$ from Eq. (12) by substituting (11) we obtain the classical wave equation for the pressure: $$ p_{xx} - (1/c_0^2)p_{tt}=0$$. It admits a general analytical (d’Alembert’s) solution, representing a sum of two waves preserving their shape and propagating in the opposite directions, i.e.

$$\begin{aligned} p(x,t)&=p_{f}(t-x/c_{0})+p_{b}(t+x/c_{0}) \\ u(x,t)&=\frac{1}{\rho c_{0}}p_{f}(t-x/c_{0})-\frac{1}{\rho c_{0}} p_{b}(t+x/c_{0}). \end{aligned}$$

Here the forward $p_f$ and backward $p_b$ waves are:

14 $$\begin{aligned} p_{f}&=\frac{1}{2}\left( p+\rho c_{0}u\right) , \qquad p_{b} =\frac{1}{2}\left( p-\rho c_{0}u\right) . \end{aligned}$$

In a real cardiovascular system, the tube is inhomogeneous: $A_0 = A_0(x)$ and $c_0 = c_0(x)$, and the equation for the pressure is more complicated, $p_{xx}+(A_x/A)p_x - (1/c^2)p_{tt}=0$, which only holds analytical solutions for a limited number of cases.

### The aneurysm as a localized reflector

Note that typically the in vivo experimental wave shapes are given in terms of up to 20 harmonic components. If the period of the main harmonic is approximately 1 s, then the wavelength of the 20th harmonic is approximately 1 m, which is greater than the length of a typical aneurysm. Such long waves can sense the aneurysm as a localized inhomogeneity (0D object) characterized by a lumped parameter: its compliance. We can therefore introduce an excess of the vessel compliance caused by the aneurysm

15 $$\begin{aligned} \Delta C_A = C_A - C_v = \frac{dV_A}{dp} - \frac{dV_v}{dp}, \end{aligned}$$

which acts as an indicator for detecting artificial or real aneurysms. In the above equation, $C_A = dV_A/dp$ is the compliance of the vessel with the aneurysm, $C_v = dV_v/dp$ is the healthy vessel compliance, $V_A$ is the volume of the vessel with the aneurysm, and $V_v$ is the volume of an equivalent healthy vessel. Thus, $\Delta C_A$ is an additional vessel compliance caused by presence of an aneurysm. Hereinafter, we will call parameter $\Delta C_A$ the additional aneurysm compliance.

The additional aneurysm compliance, $\Delta C_A$, can be expressed through the cross-sectional compliance of the vessel wall $\tilde{C}(x)$

16 $$\begin{aligned} \Delta C_A = \int _0^{L_A} (\tilde{C}_A(x) -\tilde{C}_v)\,dx \end{aligned}$$

where $L_A$ is the aneurysm length and the *x*-coordinate is referenced from the aneurysm inlet. The cross-sectional compliance is defined as

17 $$\begin{aligned} \tilde{C}(x) = \frac{dA}{dp} = \frac{2\sqrt{A}}{\beta } = \frac{2\pi a^3(x)}{E'(x)h(x)} \end{aligned}$$

where $a(x) =\sqrt{A_0/\pi }$ is the lumen radius. Substituting (17) into (16) we obtain

18 $$\begin{aligned} \Delta C_A = \int _0^{L_A} \bigg (\frac{2\pi a_A^3(x)}{E'_A(x)h_A(x)} - \frac{2\pi a_v^3}{E'_vh_v}\bigg )\,dx \end{aligned}$$

where $a_A(x)$ and $a_v(x)$ are the radii of the lumen of the vessel with and without aneurysm, respectively; $E'_A(x)$ and $h_A(x)$ are, respectively, the elastic module and wall thickness of the aneurysm; $E'_v$ and $h_v$ are the same parameters for the healthy vessel.

The artificial aneurysms used in the experiments carried out are designed to have a constant wall stiffness coinciding the wall stiffness of main tube in which the aneurysm is embedded. Substituting $E'_Ah_A=const=E'_vh_v$ into (18) we obtain the simplified expression which we will use in this work

19 $$\begin{aligned} \Delta C_A = \frac{2\pi }{E'h} \int _0^{L_A} \big (a_A^3(x){-} a_v^3\big )dx. \end{aligned}$$

and which we rewrite in the following form

20 $$\begin{aligned} \Delta C_A = \frac{2\pi a_v^3 L_A}{E'h}K,\quad K = \frac{1}{L_A}\int _0^{L_A} \bigg (\frac{a_A^3(x)}{a_v^3} - 1\bigg )\,dx. \end{aligned}$$

The non-dimensional *K* parameter is the ratio of the additional aneurysm compliance to that of the compliance of the healthy vessel of same length. It can easily be calculated numerically, but for the model aneurysms used in the experiment it can be calculated analytically as well by directly integrating Eq. (20). Both calculations give the same values as displayed in Table 1 for different aneurysm diameters (*D*) studied here. We use these values as reference ones. They are compared below with the values of *K* evaluated from the waveform analysis.

**Table 1:** Comparison of reference parameters with parameters obtained by fitting $p_f*R$ into numerical and experimental waveforms

	*D* (mm)	24 mm	34 mm	44 mm	50 mm
$\Delta t,$ ms	Refer.	57.9	57.9	57.9	57.9
	Numer.	57.4	56.8	56.6	56.5
	Exper.	61.8	52.0	53.1	55.6
$x_A,$ cm	Refer.	55.0	55.0	55.0	55.0
	Numer.	54.5	53.9	53.8	53.6
	Exper.	58.7	49.4	50.5	52.8
$\tau$, ms	Refer.	2.62	9.29	20.5	30.0
	Numer.	3.98	11.4	26.2	40.6
	Exper.	4.59	20.8	41.6	45.8
*K*, ms	Refer.	1.11	3.92	8.66	12.7
	Numer.	1.68	4.82	11.1	17.2
	Exper.	1.94	8.79	17.6	19.3
$\Delta C_A, \frac{{\mathrm{cm}}^3}{{\mathrm{MPa}}}$	Refer.	6.27	22.2	49.0	71.6
	Numer.	9.50	27.3	62.6	97.1
	Exper.	11.0	49.7	99.3	109

The reference parameters are obtained from direct measurements and calculations via Eqs. (25) and (20)

### Reflected waves from an aneurysm

Now we consider the wave reflection from an aneurysm by treating it as a 0D object with compliance $\Delta C_A$. As the wave changes its shape after the reflection, we consider first the reflection of a harmonic wave having an angular frequency, $\omega$, and a wavenumber, $k = \omega /c_0$.

Consider an infinitely long tube and an aneurysm located at distance $x_A$ from the origin (left side). From the left side of the aneurysm there will be two waves: the incident wave propagating to the right $\exp \{ikx-i\omega t\}$ (let it have an amplitude of unity) and the reflected wave $R\exp \{-ikx-i\omega t\}$, where *R* is the complex reflection coefficient that needs to be determined. To the right (exit), there will be a propagating wave $S\exp \{ikx-i\omega t\}$ with the unknown amplitude *S*. Omitting the common factor $\exp \{-i\omega t\}$, we can write the solutions as

21 $$\begin{aligned} p_1&= e^{ikx} + Re^{-ikx}\qquad \qquad \;\,\, p_2 = S e^{ikx} \end{aligned}$$

22 $$\begin{aligned} Q_1&= \frac{A}{\rho c}\Big (e^{ikx} - Re^{-ikx}\Big )\qquad Q_2 = \frac{A}{\rho c}Se^{ikx} \end{aligned}$$

where subscripts 1 and 2 correspond to the tube part before and after the aneurysm, respectively. At the point $x = x_A$ they should satisfy the matching conditions

23 $$\begin{aligned} p_1 = p_2,\quad Q_1 - Q_2 = \frac{dV}{dt} = \frac{dV}{dp}\frac{dp}{dt} = \Delta C_A (-i\omega p). \end{aligned}$$

The first condition is the continuity of the pressure across the aneurysm, and the second one indicates that the difference of the flow rates is compensated by the aneurysm volume change per unit of time. Substituting Eqs. (21) and (22) into (23) we obtain equations with respect to unknown amplitudes *R* and *S*. Solving Eqs. (21) and (22) reflection coefficient *R* can be written in the following form

24 $$\begin{aligned} R(\omega ) = \frac{i\omega \tau }{1-i\omega \tau }e^{i\omega \Delta t}. \end{aligned}$$

in which all the parameters are grouped into two variables $\Delta t$ and $\tau$ having dimension of time:

25 $$\begin{aligned} \Delta t = \frac{2x_A}{c_0} \text{ and } \tau = \frac{\rho c_0}{2A_0}\Delta C_A = \frac{L_A}{2c_0}K = \tau _0 K. \end{aligned}$$

Here $\Delta t$ is the time for the wave to travel from the origin to the centre of the aneurysm and back, and $\tau _0 = (L_A/2)/c_0$ is the time for the wave to propagate over a distance equal to the half-length of the aneurysm. For the experiments carried out, $\tau _0 \approx 2.3$ ms, $\Delta t \approx 58$ ms.

Applying the Fourier transform to the incident pulse $p_f(t)$:

26 $$\begin{aligned} p_f(\omega ) = \int _{-\infty }^{+\infty }p_f(t)e^{+i\omega t}dt \end{aligned}$$

we can express the reflected pulse through the inverse Fourier transform:

27 $$\begin{aligned} p_b(t) = \frac{1}{2\pi }\int _{-\infty }^{+\infty }p_f(\omega )R(\omega )e^{-i\omega t}d\omega . \end{aligned}$$

Performing the inverse Fourier transform (or the inverse Laplace transform after substitution $s=-i\omega$) from (24), we obtain the following function

28 $$\begin{aligned} R(t;\tau ,\Delta t) = -\delta (t-\Delta t) + H(t-\Delta t)\frac{1}{\tau }e^{-(t-\Delta t)/\tau } \end{aligned}$$

where $\delta (t)$ is Dirac’s delta function and *H*(*t*) is the unit-step Heaviside function. This allows the computation of the reflected pulse directly in the time domain through the convolution,

29 $$\begin{aligned} p_b(t) = p_f(t)*R(t) \equiv \int _{-\infty }^{t-\Delta t} p_f(t')R(t-t')\,dt'. \end{aligned}$$

If pulse $p_f(t)$ starts at instant $t=0$, then the measured waveform can be split into a few stages. At the time interval $0\le t\le \Delta t$, i.e. until the pulse reflected from the aneurysm arrives (recall $\Delta t = 2 x_A/c_0$), only the wave generated by the pump, $p_f$, can be registered. Beginning with the instant $t=\Delta t$ until that time when a pulse reflected from distal inhomogeneities (from the second fitting in our case) arrives, the measured waveform will contain the incident pulse $p_f$ and the reflected wave from the aneurysm pulse. If aneurysm parameters are unknown, they can be determined by a best fitting (by the least square method, for example) of the reflected pulse calculated by Eq. (29).

Note that the time-domain computation (despite it is much more cumbersome) has advantages over the frequency domain due to the fact that in the time domain the pulses reflected from different objects can be easily separated as they have different arrival times. In the frequency domain all the reflections are mixed together that complicates the analysis and parameter determination.

**Fig. 7:**
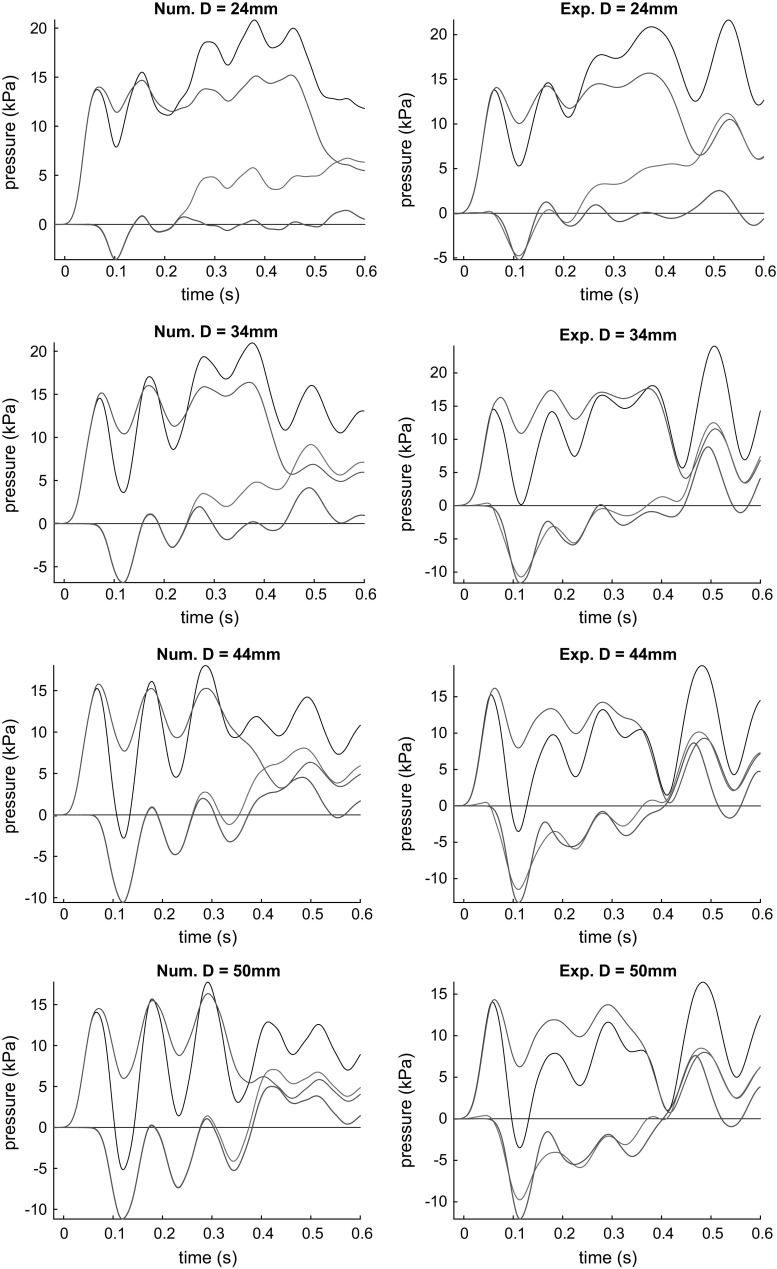
Results of separation the waveform into the forward and backward waves. *Black* pressure waveform *p*(*t*); *red* forward wave $p_f$; *blue* backward wave $p_b$; *magenta* the least square fitting of convolution $p_r(t) = p_f(t)*R(t)$

Results of wave separation performed at the location 2 cm from the inlet are shown in Fig. 7 for both the numerical and experimental data. Here, the results for the least squares fit of the $p_f(t)*R(t)$ function are also presented. Parameters $\tau$ and $\Delta t$ extracted from the fit are displayed in Table 1. In this table, one can also find other parameters calculated via $\Delta t$: distance from the  2 cm location to the midpoint of the aneurysm (should be $x_A = 55$ cm) and also parameters calculated via $\tau$: the dimensionless parameter *K* (see (20)) and the aneurysm compliance measured in $\hbox {cm}^3$ per MPa.

Thus, the proposed method allows for detecting both the aneurysm location and its compliance. The accuracy of the procedure can be seen from the comparison of reference values, which are denoted as “Refer.” in Table 1, with the results following from the signal processing procedure. One can see from Table 1 that the distance to the aneurysm can be determined rather accurately from the described fitting procedure applied to both the numerical and experimental data. As for the aneurysm compliance, the procedure gives enhanced values, in some cases more than two times higher than the analytical values. The discrepancy is greater for the experimental results due to unavailability of precise values of elastic properties of the model aneurysms. The discrepancy between analytical and numerical data is less clear and needs additional analysis. Note that the fitting procedure is sensible to the wave separation and noise.

## Application to human arterial system model

### The model

Now we apply the proposed method of aneurysm detection to a human arterial network that is modelled numerically. For the modelling, we use the arterial network ADAN 55 proposed in the work by Blanco et al. (2015) and thoroughly described in the work by Boileau et al. (2015) with some minor modifications in the segments containing the Abdominal Aorta. It comprises 55 arteries and 77 arterial segments. A small part of the ADAN 55 network is presented in Table 2 where the monitored segments are shown, i.e. segments in which pressure and velocity waveforms are outputted for the analysis. These are the segments comprising the aorta and also left and right common carotids. The segment numbering is taken from (Boileau et al. 2015). The segments with modified length (adjusted to model a real size AAA) are highlighted by an asterisk. The segment parameters necessary for our study are also listed in Table 2.

**Table 2:** Segments of the human arterial network selected for monitoring: *L* is the segment length, $D_1,D_2$ are the segment inlet and outlet diameters, and $c_0$ is the averaged wave speed

$\hbox {N}^\circ$	Name	*L*,cm	$D_1,\hbox {mm}$	$D_2,\hbox {mm}$	$c_0,\hbox {m}/\hbox {s}$
1	Aortic Arch I	7.44	31.9	25.9	4.03
3	Aortic Arch II	0.96	25.9	25.1	4.08
15	Aortic Arch III	0.70	25.1	24.6	4.09
19a	Aortic Arch IV	4.31	24.6	21.1	4.12
19b	Thoracic Aorta I	0.99	21.1	20.7	4.15
27	Thoracic Aorta II	0.79	20.7	20.4	4.15
29	Thoracic Aorta III	1.56	20.4	19.8	4.16
31	Thoracic Aorta IV	0.53	19.8	19.6	4.17
33a	Thoracic Aorta V	12.16	19.6	15.1	4.23
33b	Thoracic Aorta VI	0.32	15.1	15.0	4.30
35	Abdominal Aorta I	1.40	15.0	14.6	4.31
41	Abdominal Aorta II	0.43	14.6	14.5	4.32
43	Abdominal Aorta III	1.20	14.5	14.2	4.32
45	Abdominal Aorta IV	$10.60^*$	14.2	12.9	4.36
47	Abdominal Aorta V	$1.00^*$	12.9	11.8	4.43
5	R. Common Carotid	8.12	9.0	6.7	4.89
14	L. Common Carotid	12.13	9.0	6.7	4.89

Segments with modified length are marked by asterisk. Segment numbering is taken from (Boileau et al. 2015)

The wall thickness is determined from the relation (Blanco et al. 2015)

30 $$\begin{aligned} h = a_0\big (0.2802e^{-5.053a_0} + 0.1324e^{-0.1114a_0}\big ) \end{aligned}$$

where the local unperturbed radius $a_0(x)$ in every segment is determined via linear interpolation between inlet $a_1=D_1/2$ and outlet $a_2=D_2/2$ radii. Young’s modulus of the arterial system is assumed to be constant for the entire network: $E = 225$ kPa; the Poisson ratio, $\sigma =0.5$ as the wall material is regarded as incompressible; the standard blood parameters used are: density $\rho = 1.04$ $\hbox {g/cm}^3$, blood viscosity $\mu = 4.0$ mPa s (Boileau et al. 2015). The parameters $E,\sigma ,\rho ,h(x),a_0(x)$ enable the calculation of local (unperturbed) wave speed $c_0(x)$ and the wave speed averaged over a segment (listed in Table 2).

**Fig. 8:**
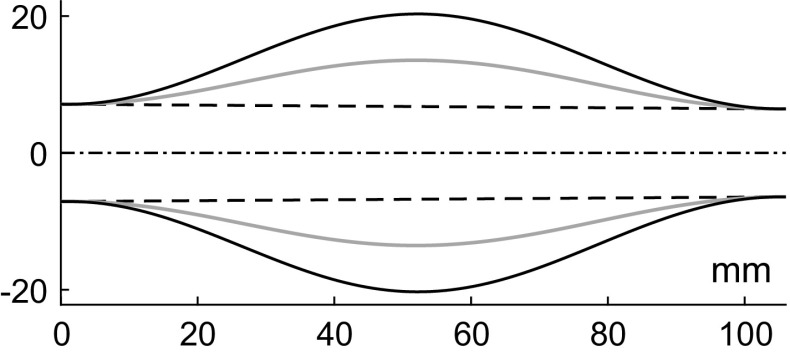
Aneurysms used in human arterial network modelling: AAA-2 (*grey*), AAA-3 (*black*). The *dashed line* indicates the healthy vessel

Segment 45 (Abdominal Aorta IV) is chosen to model the AAA. Aneurysms of two sizes are modelled: two and three times wider than the healthy vessel in its widest part. We will refer to them as AAA-2 and AAA-3, respectively. The shape is assumed to be the same as in the work by Low et al. (2012), i.e.

31 $$\begin{aligned} a(x)&= a_0(x) + \Delta a(x)\,\frac{1}{2}\left( 1 - \cos \left( 2\pi \frac{x-x_1}{L_A}\right) \right) \nonumber \\ x\in {}&[x_1,x_2] \end{aligned}$$

where $a_0(x) = a_1 + (a_2-a_1)(x-x_1)/L_s$ is the radius of the healthy vessel; $L=10.6$ cm is the segment length, $L_A=10.4$ cm is the aneurysm length; $x_1 = (L-L_A)/2 = 1$ mm and $x_2 = x_1+L_A$ are, respectively, the aneurysm start and end points; and $\Delta a(x)$ is the radius increment: $\Delta a(x) = a_0(x)$ for AAA-2 and $\Delta a(x) = 2\,a_0(x)$ for AAA-3. The aneurysm shape is shown in Fig. 8. The calculated parameters for AAA-2 are: $\tau =35.0$ ms, $K=2.94$, $\Delta C_A=22.3$ $\hbox {cm}^3\hbox {/MPa}$, for AAA-3: $\tau =119.2$ ms, $K=10.0$, $\Delta C_A=75.8$ $\hbox {cm}^3\hbox {/MPa}$.

**Fig. 9:**
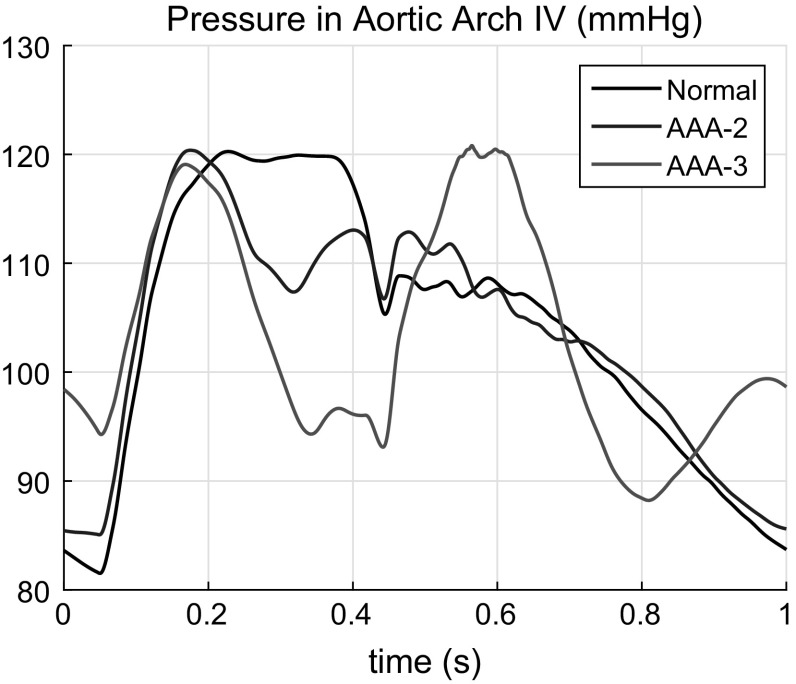
Pressure variation in Aortic Arch IV

The flow rate $Q^{\mathrm{in}}(t)$ is imposed at the inlet of the first segment as in (Blanco et al. 2015; Boileau et al. 2015) with the heartbeat period of $T = 1$ s. The three-element Windkessel (lumped) model is applied to all terminal arteries. The simulation runs for three cycles to generate a periodic solution, which takes about 3 min. The third cycle is used for the analysis. The pressure *p*(*t*), average velocity *u*(*t*) and area variation *A*(*t*) are monitored at the centre of the segments indicated in Table 2 (aorta and left and right common carotids). An example of the computed pressure waveform in segment 19a (Aortic Arch IV) is shown in Fig. 9.

As seen, the presence of an aneurysm results in a pressure drop at a certain time interval after the main peak. This is particularly pronounced in the case of the AAA-3 aneurysm. The graphs clearly show the main feature of the large AAA’s presence, i.e. a pressure drop just after the main peak followed by a distinct second peak after that.

### AAA detection based on aortic waveform analysis

We apply the proposed aneurysm detection method to the sites located in aortic segments. First, eight segments listed in Table 2 are selected for that: from 1 to 31 (from Aortic Arch I to Thoracic Aorta IV). Equation (14) is applied to separate the forward $p_f$ and backward $p_b$ waves. Then the convolution $p_f(t)*R(t)$ is calculated. This function has to be fitted into the backward wave $p_b$ via the least squares method. In the experimental and numerical modelling described in previous sections, the analysis was easier when the pulses reflected from the aneurysm and other parts of the network are separated in time. As a result, the pulse reflected from the aneurysm was the first to reach the monitoring point as a backward wave. In the case of a full human arterial network, however, the pressure pulse exerts multiple reflections from bifurcations proximal to the aneurysm, and, hence, there is no such time interval where the only pulse reflected from the aneurysm exists. The reflections here are mixed with other reflections. Therefore, the aneurysm detection is more complicated, and the aneurysm detection procedure has to be modified.

According to Eq. (24), the reflection coefficient is small for low frequencies. Hence, the signal reflected from the aneurysm will not contain the lowest harmonic components. So it is useful to filter the lowest frequency components as they are likely caused by other parts of the network rather than by the AAA. We perform the high-pass filtering by employing Gaussian smoothing, i.e.

32 $$\begin{aligned} p'_b = p_b - G*p_b\quad \text{ where } G=\frac{\exp \{-(t/\delta )^2\}}{\sqrt{\pi }\delta }. \end{aligned}$$

Here $\delta$ is the Gaussian function width and $\delta \approx 6\tau$ gives a suitable result where an expected value of $\tau$ is used, but note that the procedure is not sensitive to this parameter.

Another challenge is that the background noise of the waves reflected from locations other than the AAA remains. Therefore, we fit the function $p_r = p_f*R + B$ into the $p'_b$ function where *B* is the parameter to be found. The *B* parameter approximates the background of the remaining reflections, noise and the forward wave pulse. A simple constant value for this parameter is sufficient here.

Now we minimize the following functional with respect to the three parameters, $\tau$, $\Delta t$ and *B*:

33 $$\begin{aligned} \int _{t_1}^{t_2}(p'_b - p_r)^2dt \rightarrow \min \end{aligned}$$

where $t_1=t_0 + \Delta t$ equals the sum of the pulse starting instant $t_0$ and the expected time of propagation from the site to the aneurysm and back $\Delta t$, $t_2 = t_1 + T/3$. The choice of *T* / 3 for the width of the interval gives satisfactory results in all the cases considered. Note that the procedure is more sensitive to the time interval $[t_1,t_2]$.

**Fig. 10:**
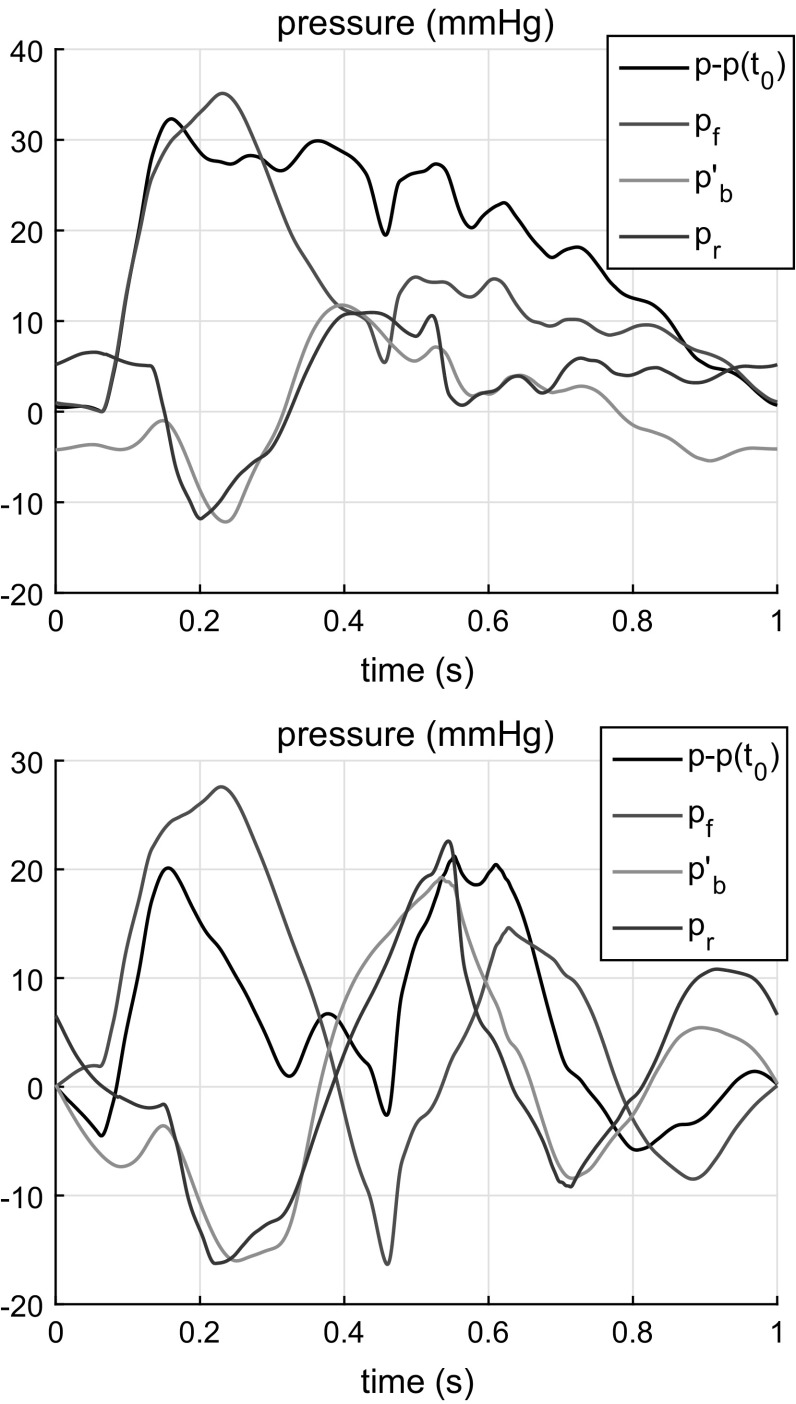
Results of fitting reflected waveform for model AAA-2 (*top*) and AAA-3 (*bottom*) for site located in Aortic Arch IV. *Black* pressure waveform $p(t)-p(t_0)$; *Red* forward wave $p_f$; *Blue* backward filtered wave $p'_b$; *Magenta* the least square fitting of the function $p_r(t) = p_f(t)*R(t)+B$

Examples of the waveform fit for segment 19a (Aortic Arch IV) are shown in Fig. 10.

The results of function fitting for all eight monitored segments are listed in Table 3. The results show that the determination of the aneurysm parameters, its additional compliance $\Delta C$ and time lag $\Delta t$, is quite possible for the human network as well. The accuracy can be estimated by comparing $\Delta t$ in the 3rd and 5th columns with the $\Delta t^\mathrm{ref}$ in the second column. It is also can be estimated by comparing $\Delta C$ in the 4th and 6th column with that indicated in the second raw of Table 3. The accuracy is not perfect but still provides a correct order of magnitude for the value. Note that the value of aneurysm parameter $\Delta C$ depends monotonically on the position of the monitoring point: the closer it is to the AAA—the greater the value of $\Delta C$. The exact value of the $\Delta C$ parameter is obtained in segment 15 (Aortic Arch III) for both the AAA-2 and AAA-3. For the proximal sites to that segment, the procedure underestimates the $\tau$ value and for the distal sites overestimates it. Similar results are also obtained for the time lag $\Delta t$, which allows for the calculation of the distance to the AAA. Accuracy in finding $\Delta t$ becomes noticeably worse for the larger aneurysm.

**Table 3:** Comparison of the reference parameters of a model AAA and parameters evaluated through waveform analysis: $\Delta t$, ms and $\Delta C_A$, $\hbox {cm}^3/\hbox {MPa}$

$\hbox {N}^\circ$	$\Delta t^{\mathrm{ref}}$	AAA-2		AAA-3	
		$\Delta C_A^{\mathrm{ref}}{=}22.3$		$\Delta C_A^{\mathrm{ref}}{=}75.8$	
		$\Delta t$	$\Delta C_A$	$\Delta t$	$\Delta C_A$
1	163	178	13	220	48
3	143	137	19	179	58
15	139	126	21	163	72
19a	126	105	26	132	90
19b	113	90	32	109	83
27	109	84	33	103	83
29	104	76	35	95	84
31	99	69	38	87	86
5	194	151	13	172	48
14	189	145	16	161	71

The reference propagation time $\Delta t^{{\mathrm{ref}}}$ is calculated based on average segment wave speed given in Table 2. The reference values of the aneurysm compliance for AAA-2 and AAA-3 calculated via Eq. 19 are given in the top of the table. Results of velocity wave fitting in carotids (segments 5 and 14) are shown in the last two rows of the table

**Fig. 11:**
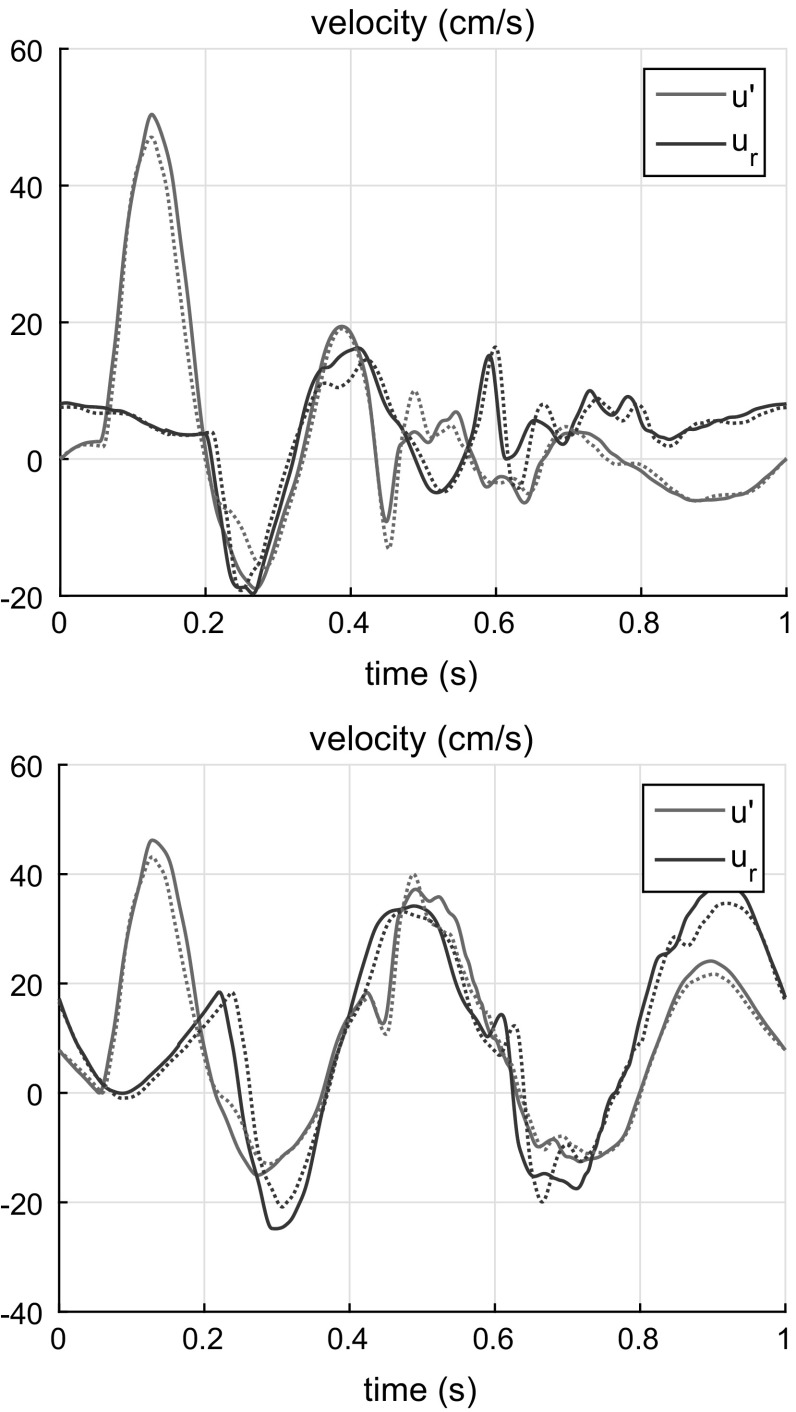
Results of fitting reflected waveform for model AAA-2 (*top*) and AAA-3 (*bottom*) for site located in LCA (*solid line*) and RCA (*dotted line*). *Blue* filtered velocity wave $u'_b$; *Magenta* the least square fitting of the function $u_r(t) = u'(t)*R(t)+C$

### AAA detection based on carotid waveform analysis

Finally, we consider the case that is most relevant when targeting this technology for non-invasive assessments in clinical practice, i.e. the determination of the AAA parameters by measuring the waveforms at more accessible sites such as the carotid artery. Here we will use the waveforms computed in the middle of the right common carotid (RCA) (segment 3) and left common carotid (LCA) (segment 14). This case is more complicated because the wave separation does not help, as the forward pulse and the pulse reflected from the AAA propagate in the same direction. Therefore, aneurysm detection based on the carotid waveform requires a more sophisticated approach of signal processing. The approach should be based on the possibility to calculate the shape of pulse reflected from the aneurysm and then to recognize it on the background of the main signal, other reflections and noise. Our proposed signal processing solution to this problem is described next.

We propose to apply the above method to the velocity waveform. This makes sense as the velocity waveform peak has a shorter duration, and therefore, the reflected wave from the AAA pulse should be easier to distinguish on the background signal consisting of the direct pulse and other reflections. Thus, we take the velocity (or flow rate) waveform, filter it using $u' = u - G*u$ where *G* is given by Eq. (32) and fit the function $u_r = u'*R + B$ using least squares with respect to the same three parameters: aneurysm characteristic time $\tau$, time lag $\Delta t$ and pulse background *B*.

In the case of the LCA, the time lag is the time of pulse propagation from the LCA inlet to the AAA and back. In the case of the RCA, the time lag is the duration of pulse propagation from the brachiocephalic trunk inlet (segment 2 in (Boileau et al. 2015)) to the AAA and back. Calculated values of the time lag for these cases are listed in the second column in Table 3. The results of the least squares fit of the $u_r$ function are shown in Fig. 11. From Table 3 we see that the proposed method underestimates the $\tau$ parameter and the time lag $\Delta t$ but nevertheless gives reasonable values for the AAA parameters. Note that in the absence of the aneurysm, the method gives a value of $\Delta C < 2$ $\hbox {cm}^3\hbox {/MPa}$. This indicates the method is highly sensitive to the presence of an aneurysm.

## Discussion and conclusion

A new method is developed to detect and characterize aortic aneurysms using the pulse reflections caused by those aneurysms. An experimental set-up is used to: 1) investigate waveform changes caused by aneurysms of various severity, 2) to validate our 1D computational model in the presence of aneurysms. The results obtained allow us to develop a new method of aneurysm detection based on waveform analysis. These results are then successfully employed to a numerical model of a human arterial system to evaluate the potential for detecting aneurysms. Analysis of the waveforms observed in the carotid arteries shows that aneurysms can be detected in terms of location and severity through our new method.

The proposed method, which incorporates the reflected waveform computation $p_f*R$ or $u*R$ with the subsequent least squares fitting procedure, looks promising for aneurysm detection and determination of its main parameters. The novel parameter introduced is the aneurysm compliance as defined by Eq. (19) and can be determined via this procedure. Moreover, if we are working in the frequency range in which the 1D theory is applicable and accurate enough, the $\Delta C_A$ parameter looks to be the only parameter of an aneurysm that can be determined through the waveform analysis. To extract finer details of an aneurysm (its more detailed geometrical and elastic parameters) it is necessary to register and process the waveform in the higher frequency range where the wavelength is comparable to the aneurysm dimension. The 1D theory may be inadequate for this.

The aneurysm compliance given by Eq. (15) is a very useful parameter. As it is proportional to the integral of the vessel diameter cubed (see Eq. (19)), the wider parts of the aneurysm strongly contribute to its value. This can help to evaluate the aneurysm diameter for most of aneurysm geometrical shapes.

Note that in this work we focus on the effect of aneurysm geometries on the pulse wave reflections. Therefore, the artificial aneurysms have been produced to keep the complexities to a minimum by adapting constant thickness and stiffness. We use identical settings in the numerical simulations. If the wall thickness *h* and its elastic modulus $E'$ also vary along the vessel, then Eq. (18) should be used instead of Eq. (19).

Observe that the wall stiffness $h_A(x)E'_A(x)$ is in the denominator in (18). Therefore, the $\Delta C_A$ parameter is very sensitive to the local wall softness: the softer/thinner parts of the wall strongly contribute to the integral (18). Hence, rapid increase in time of the aneurysm compliance $\Delta C_A$ can indicate that some parts of the wall are very thin and soft. This can potentially allow aneurysm monitoring, especially when the proposed method is employed in parallel to other modalities, for example, ultrasound (US). This may provide an opportunity to evaluate the elastic parameters of an aneurysm and predict its rupture.

One of the advantages of the proposed method is that it can be implemented in the time domain where it is easier to distinguish pulses reflected from different parts in the cardiovascular system. Another advantage is that there is no need to calculate the reflection coefficient as the pulse reflected from an aneurysm definitely changes its shape. Therefore, if we define the reflection coefficient, for example, as a ratio of peak values of reflected and incident pulses, it will depend on the shape of the incident pulse, i.e. it will not be invariant with respect to incident pulse shape and parameters. The same remark can be directed to the pulse intensity analysis(Parker and Jones 1990; Khir et al. 2001; Swillens et al. 2008; Hughes and Parker 2009). In the frequency domain, at any particular frequency all the reflected pulses are mixed together, and it is difficult to outline the contribution of the aneurysm. So those approaches can at best help to detect the presence of an aneurysm, but they can hardly help to determine its geometrical/elastic properties accurately.

The results of the work are very promising and show that the proposed method has a real potential to be further developed into a powerful technique that will be adopted in a clinical setting some day.

### Limitations

In this subsection, the limitations of the proposed method of detecting aneurysms are briefly highlighted. Although the limitations are less severe when comparing the proposed model to the in vitro experimental data, the limitations become prominent when the model is applied to in vivo data of patients. This is due to the fact that the in vitro experimental parameters are controlled but the patient data come with many unknowns. Some of the specific limitations of the work are briefed below.

The ability of the model to correctly determine the aneurysm size depends on the aneurysm wall stiffness *Eh* as demonstrated by Eq. (18). Although a large number publications on aneurysm wall properties are constantly appearing in the literature, the uncertainty related to patient specificity will be extremely difficult to eliminate. The question on relationship between the aneurysm size and material property is also not completely answered with contradicting reports in the literature. For example, Sekhri et al. (2004) found that the aneurysm wall becomes stiffer with an increase in aneurysm size whilst Kolipaka et al. (2016) did not confirm this. Therefore, if the vessel wall stiffens with the an increase in aneurysm size, then the aneurysm compliance grows at a slower rate than that is described by equation (19), which will underestimate the aneurysm diameter. Moreover, if the wall stiffness grows proportionally to the cube of the aneurysm diameter, then the aneurysm compliance will remain very small and the proposed method may not detect the aneurysm. On the other hand, if the aneurysm is very compliant locally, which can occur just before the rupture, then the pronounced reflection will immediately indicate the presence of an aneurysm. In addition to these limitations, assumed parameters of the healthy vessels can also contribute to the inaccuracy in predictions.

It is also important to mention that Eqs. (24) and (28) are derived for a tube with a constant cross-sectional area and an embedded aneurysm. Such conditions can be easily reproduced using an in vitro experimental set-up. Nevertheless, for accurate aneurysm detection, these equations need generalization to the in vivo cases that have tapered blood vessels and varying vessel stiffness. We will consider such generalizations in a subsequent, future work. However, if the tapering and stiffness variations are small, then the effects of such variations on the results will be much lower than that of the uncertainties due to other parameters.

## Figures and Tables

**Fig. 12:**
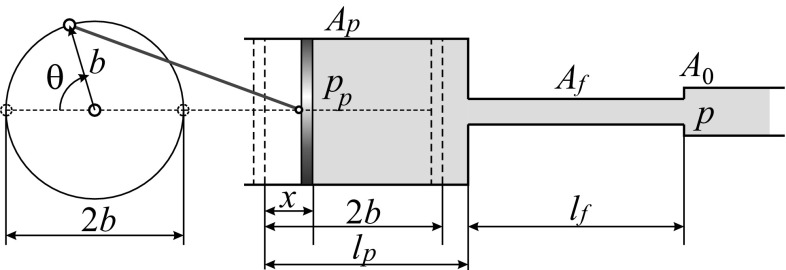
Simplified scheme of the pump, initial fitting pipe and part of the main tube

**Fig. 13:**
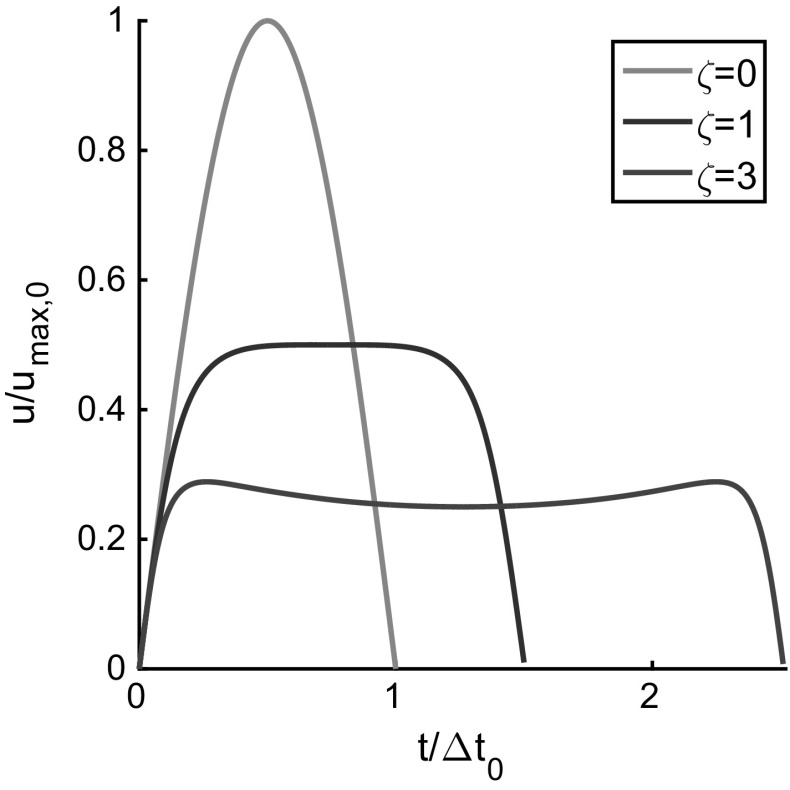
Flow velocity *u*(*t*) (normalized by the maximal velocity without load, $u_{\max ,0}$) versus time *t* (normalized by the pulse duration without load $\Delta t_0$

## Appendix 1: Numerical scheme

The numerical scheme applied to solve the equations is similar to that described in Low et al. (2012). Equations (6), (7) are discretized by adopting second-order Taylor–locally conservative Galerkin (LCG) finite element method.

$$\begin{aligned} \mathbf {U}^{n+1}= & {} \mathbf {U}^{n} + \Delta t\big ( \mathbf {S}^{n}- \mathbf {F}^{n}_x \big ) \\&+\frac{\Delta t^{2}}{2}\bigg [ \mathbf {S}_{\mathbf {U} }^{n}\big ( \mathbf {S}^{n}- \mathbf {F}^{n}_x \big )- \frac{\partial }{\partial x} \Big ( \mathbf {F}_{\mathbf {U}}^{n}\big ( \mathbf {S}^{n}- \mathbf {F}^{n}_x \big ) \Big ) \bigg ] \end{aligned}$$

where $$\displaystyle \mathbf {F}_{x}^{n}=\frac{\partial \mathbf {F}^{n}}{\partial x}$$, $$\displaystyle \mathbf {F}_{\mathbf {U}}^{n}=\frac{\partial \mathbf {F}^{n}}{\partial \mathbf {U}}$$, $$\displaystyle \mathbf {S}_{\mathbf {U}}^{n}=\frac{\partial \mathbf {S}^{n}}{\partial \mathbf {U}}$$.

The fully discrete form using LCG can be written as

34 $$\begin{aligned} \left[ \mathbf {M}_{e}\right] \left\{ \Delta \mathbf {U}\right\} ^{n}=\Delta t \Big ( \left[ \mathbf {K}_{e}\right] \left\{ \mathbf {F}\right\} ^{n}+\left[ \mathbf {L}_{e}\right] \left\{ \mathbf {S}\right\} ^{n}+\left\{ \mathbf {f} _{\varGamma _{e}}\right\} ^{n}\Big ) \end{aligned}$$

where $\left[ \mathbf {M}_{e}\right]$ , $\left[ \mathbf {K}_{e}\right]$, $\left[ \mathbf {L}_{e}\right]$ and $\left\{ \mathbf {f}_{\varGamma _{e}}\right\} ^{n}$ are the elemental mass matrix, elemental convection matrix, elemental source matrix and elemental boundary flux vector, respectively.

$$\begin{aligned} \left[ \mathbf {M}_{e}\right]&=\frac{\Delta x_{e}}{6} \begin{bmatrix} 2&1 \\ 1&2 \end{bmatrix} \\ \left[ \mathbf {K}_{e}\right]&=\left( \frac{1}{2}+\frac{\Delta t}{4}{\breve{\mathbf {S}}}_{\mathbf {U}}^{n}\right) \begin{bmatrix} -1&-1 \\ 1&1 \end{bmatrix} -\frac{\Delta t}{2\Delta x_{e}}\breve{\mathbf {F}}_{\mathbf {U}}^{n} \begin{bmatrix} 1&-1 \\ -1&1 \end{bmatrix} \\ \left[ \mathbf {L}_{e}\right]&=\frac{\Delta x_{e}}{6}\left( 1+\frac{\Delta t }{2}\breve{\mathbf {S}}_{\mathbf {U}}^{n}\right) \begin{bmatrix} 2&1 \\ 1&2 \end{bmatrix} +\frac{\Delta t}{4}{\breve{\mathbf {F}}}_{\mathbf {U}}^{n} \end{aligned}$$

where $\Delta x_{e}$ is the length of an element *e*, $\displaystyle {\breve{\mathbf {S}}}_{\mathbf {U}}^{n}$ and $\displaystyle {\breve{\mathbf {F}}}_{\mathbf {U} }^{n}$ are the average values over the elemental subdomain. Vector $\left\{ \mathbf {f}_{\varGamma _{e}}\right\} ^{n}$ is used to link information between subdomains, which for the element *e* bounded by nodes *i* and *j* takes the form

35 $$\begin{aligned} \left\{ \mathbf {f}_{\varGamma _{e}}\right\} ^{n}= \begin{Bmatrix} \widetilde{{\bar{\mathbf {F}}}}_{i}^{n} \\ -\widetilde{{\bar{\mathbf {F}}}}_{j}^{n} \end{Bmatrix} \end{aligned}$$

where $\widetilde{{\bar{\mathbf {F}}}}$ is the average value of ${\hat{\mathbf {F}}}$ from adjacent elements with ${\hat{\mathbf {F}}}$ representing the computed boundary flux. It is computed in a small post-processing step.

Equation (34) is solved individually and independently over the individual elements. However, this will result in multiple solutions for each node, which is non-unique. To achieve a unique and continuous solution throughout the domain, the values of adjacent elements are averaged. This continuous solution can then be used for computing the numerical boundary flux of Eq. (35) for the following time step in a small post-processing step. Note that for elements at a global boundary, which are situated at both ends of each vessel segment, boundary conditions need to be specified for $\left\{ \mathbf {f} _{\varGamma _{e}}\right\}$ as there is no adjacent element. The stability criterion used in the current study is given as (Mynard and Nithiarasu 2008; Low et al. 2012)

$$\begin{aligned} \Delta t \le 0.9 \min _e \frac{\Delta x_e}{(\lambda _f)_e} \end{aligned}$$

where $(\lambda _f)_e$ is the elemental forward eigenvalue.

## Appendix 2: Initial pulse

Consider a simplified scheme of the pump shown in Fig. 12. If the radius of rotation of the joint between the wheel and the crank is *b*, then the piston stroke is 2*b*. If the current angle of the wheel $\theta$ and the current piston position *x* are $x = b\,(1-\cos \theta )$, then the piston $u_p$ velocity equals $u_p \equiv \dot{x} =\dot{\theta }\,b\,\sin \theta$. Let the cross-sectional area of the piston, fitting pipe and the main tube be $A_p$, $A_f$, $A_0$, respectively, then the velocity in the fitting pipe will be $u_f = (A_p/A_f)u_p$, and velocity in the inlet of the main tube will be $u = (A_p/A_0)u_p$. If we neglect the reflection from the aneurysm, second fitting and others, then only the out going wave will be generated in the main tube. Hence, if we neglect the nonlinear and viscous effects, then the inlet pressure will be

36 $$\begin{aligned} p = \rho c \frac{A_p}{A_0} u_p. \end{aligned}$$

The pressure at the inlet of the fitting will be $p + \rho \,(A_p/A_f)\,l_f \ddot{x}$, whereas the pressure at the piston $p_p = p + \rho \,(A_p/A_f)\,l_f\ddot{x} + \rho \, (l_p - x)\,\ddot{x}$, where $\ddot{x} = \ddot{\theta }\,b\sin \theta + (\dot{\theta })^2b\cos \theta$ is the piston acceleration, $l_p$ is the cylinder length: $l_p\ge 2b$.

The piston acts with the force $F_p = A_p p_p$ and produces the moment $M = F_p l$ where $l = b\sin \theta$ is the length of the lever arm. If the engine produces a constant torque *T*, the moment balance equation reads as

37 $$\begin{aligned} T = f\dot{\theta } + M \end{aligned}$$

where *f* is the friction coefficient. Here we neglected the piston and the crank mass compared to the mass of the moving liquid. The second term on the RHS of (37) describes the load from the liquid on to the engine.

In the absence of the liquid load ($M = 0$), the engine would rotate uniformly with the constant angular velocity

38 $$\begin{aligned} \varOmega \equiv \dot{\theta } = \frac{T}{f}. \end{aligned}$$

Therefore, the duration of the positive (or negative) pulse will be equal to the half-period of the wheel rotation

39 $$\begin{aligned} \Delta t_0 = \frac{\pi }{\varOmega } = \frac{\pi f}{T}. \end{aligned}$$

The velocity in the inlet of the main tube would be

40 $$\begin{aligned} u_0 = u_{\max ,0}\sin (\varOmega t),\quad \hbox {where }u_{\max ,0} = \frac{A_p}{A_0}\,b\,\varOmega . \end{aligned}$$

In the presence of the load pulse becomes wider and its maximal velocity becomes lower. To account qualitatively for influence of the liquid load, we neglect inertia of the liquid in the cylinder and the fitting pipe. Then obtain the first-order ODE

41 $$\begin{aligned} T = f\dot{\theta } + \rho c \frac{A_p^2}{A_0}b^2\sin ^2\theta \,\dot{\theta } \end{aligned}$$

which can be rewritten in the form with minimal number of parameters:

42 $$\begin{aligned} \dot{\theta }\, \left( 1+\zeta \sin ^{2}\theta \right) =\varOmega , \qquad \zeta = \frac{\rho c\, A_p^2\,b^2}{A_0 f} \end{aligned}$$

Dimensionless parameter $\zeta$ characterizes the fluid load on the engine.

The results of numerical integration are plotted in Fig. 13. Here the waveform becomes two humped if the load from the piston tube system is high enough. Accounting for more parameters of the set-up makes the waveform more complicated and makes the humps non-symmetrical.
